# Validating the accuracy of deep learning for the diagnosis of pneumonia on chest x-ray against a robust multimodal reference diagnosis: a post hoc analysis of two prospective studies

**DOI:** 10.1186/s41747-023-00416-y

**Published:** 2024-02-02

**Authors:** Jeremy Hofmeister, Nicolas Garin, Xavier Montet, Max Scheffler, Alexandra Platon, Pierre-Alexandre Poletti, Jérôme Stirnemann, Marie-Pierre Debray, Yann-Erick Claessens, Xavier Duval, Virginie Prendki

**Affiliations:** 1 Department of Diagnostics, Geneva University Hospitals, Geneva, Switzerland; 2 Division of Internal Medicine, Riviera Chablais Hospital, Rennaz, Switzerland; 3 Department of Medicine, Geneva University Hospitals, Geneva, Switzerland; 4 Department of Radiology, APHP, Hôpital Bichat, University Paris Cité, Inserm UMR1152, Paris, France; 5 Department of Emergency Medicine, Centre Hospitalier Princesse Grace, La Colle, Principality of Monaco Monaco; 6 Department of Epidemiology and Clinical ResearchInserm CIC 1425UMR 1138, APHP, Hôpital BichatUniversity Paris CitéIAME, Paris, France; 7 Department of Rehabilitation and Geriatrics, Geneva University Hospitals, Geneva, Switzerland; 8 Division of Infectious Disease, Geneva University Hospital, 4 Rue Gabrielle Perret-Gentil, 1211 Geneva 14, Switzerland

**Keywords:** Artificial intelligence, Chest x-ray, Deep learning, Diagnosis, Pneumonia

## Abstract

**Background:**

Artificial intelligence (AI) seems promising in diagnosing pneumonia on chest x-rays (CXR), but deep learning (DL) algorithms have primarily been compared with radiologists, whose diagnosis can be not completely accurate. Therefore, we evaluated the accuracy of DL in diagnosing pneumonia on CXR using a more robust reference diagnosis.

**Methods:**

We trained a DL convolutional neural network model to diagnose pneumonia and evaluated its accuracy in two prospective pneumonia cohorts including 430 patients, for whom the reference diagnosis was determined a posteriori by a multidisciplinary expert panel using multimodal data. The performance of the DL model was compared with that of senior radiologists and emergency physicians reviewing CXRs and that of radiologists reviewing computed tomography (CT) performed concomitantly.

**Results:**

Radiologists and DL showed a similar accuracy on CXR for both cohorts (*p* ≥ 0.269): cohort 1, radiologist 1 75.5% (95% confidence interval 69.1–80.9), radiologist 2 71.0% (64.4–76.8), DL 71.0% (64.4–76.8); cohort 2, radiologist 70.9% (64.7–76.4), DL 72.6% (66.5–78.0). The accuracy of radiologists and DL was significantly higher (*p* ≤ 0.022) than that of emergency physicians (cohort 1 64.0% [57.1–70.3], cohort 2 63.0% [55.6–69.0]). Accuracy was significantly higher for CT (cohort 1 79.0% [72.8–84.1], cohort 2 89.6% [84.9–92.9]) than for CXR readers including radiologists, clinicians, and DL (all *p*-values < 0.001).

**Conclusions:**

When compared with a robust reference diagnosis, the performance of AI models to identify pneumonia on CXRs was inferior than previously reported but similar to that of radiologists and better than that of emergency physicians.

**Relevance statement:**

The clinical relevance of AI models for pneumonia diagnosis may have been overestimated. AI models should be benchmarked against robust reference multimodal diagnosis to avoid overestimating its performance.

**Trial registration:**

NCT02467192, and NCT01574066.

**Key point:**

• We evaluated an openly-access convolutional neural network (CNN) model to diagnose pneumonia on CXRs.

• CNN was validated against a strong multimodal reference diagnosis.

• In our study, the CNN performance (area under the receiver operating characteristics curve 0.74) was lower than that previously reported when validated against radiologists’ diagnosis (0.99 in a recent meta-analysis).

• The CNN performance was significantly higher than emergency physicians’ (*p* ≤ 0.022) and comparable to that of board-certified radiologists (*p* ≥ 0.269).

**Graphical Abstract:**

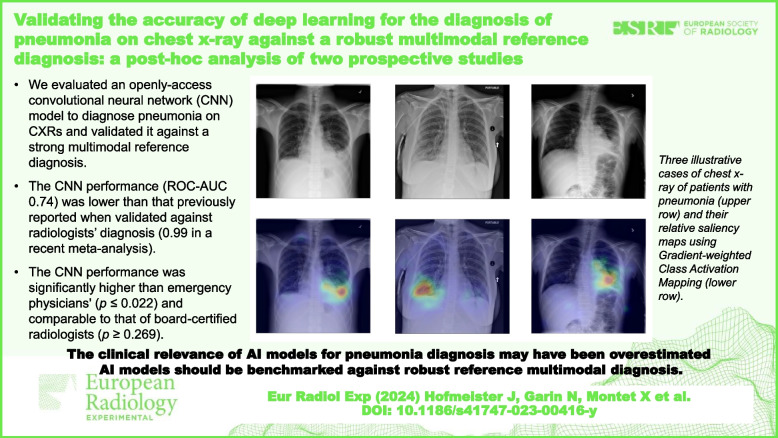

**Supplementary Information:**

The online version contains supplementary material available at 10.1186/s41747-023-00416-y.

## Background

Lower respiratory tract infections are among the most common infections and are a leading cause of death globally [1, 2]. A diagnosis of pneumonia is suggested by clinical findings but always requires confirmation on imaging studies, primarily chest x-ray radiography (CXR) [3–5]. While CXR is widely available, its interpretation is time-consuming and may lead to important interobserver variability among radiologists and clinicians [6–10]. Moreover, the detection of pneumonia on CXR requires experienced radiologists who are not always readily available in an emergency setting.

Deep learning (DL) has become very popular and helpful in various medical diagnoses. Many artificial intelligence (AI) tools have been developed to diagnose pneumonia on CXRs [11–18], notably to overcome the lack of experienced reviewers in an emergency context. The reference diagnosis used to assess their performance is commonly based on radiologists’ interpretation of the CXR, despite the poor sensitivity of CXRs and a low interobserver agreement [6, 7, 10]. The performance of AI in the diagnosis of pneumonia on CXR may thus be biased and overestimated because of the inaccuracy of the reference diagnosis commonly used in the literature. Moreover, the performance of AI has rarely been compared to other imaging modalities increasingly used for the diagnosis of pneumonia, such as thoracic CT scan, which has better accuracy than CXR.

Against this background, we developed a convolutional neural network (CNN) DL model to diagnose pneumonia on a large cohort from public datasets and compare it to a strong reference diagnosis. We aimed to compare its performance for the diagnosis of pneumonia on CXR with those of (1) emergency physicians and (2) senior radiologists reading the same CXR, as well as (3) radiologists interpreting a CT scan performed concomitantly, using two prospective observational cohorts of patients with suspected pneumonia which used a panel of experts for the reference diagnosis. The CNN model used in this article is made publicly available online to the scientific community.

## Methods

### Training cohort

We first trained a CNN system to identify pneumonia on CXRs of a large cohort of patients derived from several public datasets. Our study is reported in accordance with the CLAIM checklist [19], modeled on the STARD guidelines.

Our training cohort included 700,555 frontal CXRs from the following datasets: CheXpert (*n* = 223,648) [20]; MIMIC-CXR (*n* = 371,920) [21]; and ChestXray-14 (*n* = 104,987) [22, 23]. Of the data, 85% were used for the training set (*n* = 595,472 CXRs), 10% for the validation set (*n* = 70,055 CXRs), and 5% for the testing set (*n* = 35,028 CXRs). Only CXRs with frontal views from these datasets were used, without other exclusion criteria.

All these CXRs were associated with a binarized (yes/no) diagnosis of pneumonia in order to perform the CNN training. For the CheXpert and MIMIC-CXR datasets, the pneumonia label was provided in the public datasets and included 62,298 and 67,435 cases, respectively. For the ChestXray-14 dataset, the pneumonia diagnosis was relabeled by Rajpurkar et al. [24] and included 31,851 pneumonia cases. Of note, in these datasets, the reference diagnosis was the interpretation of CXR reported in routine by radiologists. All images in the three datasets were resized (512 × 512 pixels) and normalized (-1 to 1) to match standard CNN training practice. We did not used other preprocessing or data augmentation methods during the CNN training.

### CNN model training

This large dataset of frontal CXRs was used to train a CNN with an EfficientNet-B4 architecture pre-trained on ImageNet and using Tensorflow (v2.11.0), which is open access [13]. The last layer of the EfficientNet-B4 CNN was replaced by a dense layer of 1 neuron in order to fit our unique prediction label (*i.e.*, pneumonia), with a sigmoid function activation. This model was trained to identify pneumonia, using Adam optimizer with standard parameters [25]. We trained the CNN for 200 epochs with minibatches of size 8 and used an initial learning rate of 0.001, which was reduced by a factor of 10 each time the loss on the tuning set plateaued after 10 epochs. The best model was selected based on its performance evaluated by the area under the receiver operating characteristic curve (ROC-AUC) on the validation set (ROC-AUC = 0.988) and showed excellent performance on the internal testing set from the same large cohort (ROC-AUC = 0.985). No change in thresholding after training this model and validating and testing it on the data split from the same cohort. We used our institution’s high-performance computing system to train this network, on a node with 8 NVIDIA 3090 GPUs. The model developed for this article is publicly available on GitHub: https://github.com/jeremyhofmeister/pneumoniaCXR.

### External validation cohorts

The validation cohorts were derived from two prospective clinical studies comparing the performance of CXR and thoracic CT scan in the diagnosis of pneumonia upon hospital admission.

The “Low-dose CT for the diagnosis of pneumonia in elderly patients’ study” (PneumOld-CT, NCT02467192) [26] prospectively included 200 consecutive > 65 year-old patients hospitalized between 1 May 2015 and 30 April 2016 for suspected community-acquired pneumonia and nursing home-acquired or hospital-acquired pneumonia at Geneva University Hospitals (details in Additional file 1: Appendix 1). A frontal CXR (with or without lateral view) was obtained for all patients upon admission and interpreted by the attending emergency physician in charge of the patient, who had access to all patient’s clinical information. All CXRs were also reviewed a posteriori by two senior radiologists certified in thoracic imaging (with over 10 years of experience) (radiologist 1, radiologist 2). A low-dose CT scan without intravenous administration of contrast agent was performed upon admission, within 12 h after CXR, and was interpreted by a board-certified radiologist in charge of reviews.

The “Early Chest Computed Tomography Scan to Assist Diagnosis and Guide Treatment Decision for Suspected Community-acquired Pneumonia” study (PACSCAN, NCT01574066) [27] included 319 consecutive > 18-year-old patients with suspected community-acquired pneumonia only in the emergency units of four tertiary teaching hospitals of the Assistance Publique—Hôpitaux de Paris between November 2011 to January 2013. For each patient, a CXR was obtained according to routine clinical protocol and interpreted by the attending emergency physician in charge of the patient who also had access to the patient’s clinical and biological information. The CXR was also interpreted by a board-certified radiologist. A CT scan, which could be full-dose and enhanced with contrast, if necessary, was acquired in all patients within 4 h of inclusion in the study and reviewed by a board-certified thoracic radiologist.

In both cohorts, the probability of pneumonia assessed by emergency physicians and radiologists was reported on a Likert scale, with 3 points in the PneumOld-CT and 4 points in the PACSCAN cohorts. In order to calculate the difference in diagnostic performance, these scales were adapted to obtain a binary output of pneumonia (present/absent). For the PneumOld-CT study, intermediate and high probabilities were considered as presence and low probabilities as absence of pneumonia; in the PACSCAN study, definite and probable diagnoses were considered as pneumonia and possible and excluded diagnoses were considered as the absence of pneumonia. We repeated the analysis of the PACSCAN cohort, including possible pneumonia as part of the pneumonia group (*i.e.*, definite, probable, and possible pneumonia considered as pneumonia; and excluded pneumonia considered as non-pneumonia).

### Reference diagnosis

In both cohorts, the reference diagnosis of pneumonia was adjudicated a posteriori and in accordance with international guidelines by a panel of experts using a Delphi method; the experts had access to all available imaging, biological, and clinical data and were aware of patients’ long-term evolution [3, 26–28]. Details are described in Additional file 1: Appendix 1.

### Prediction of pneumonia by the CNN

Processing of CXRs from both cohorts was performed by one of the authors (J.H.) on a commonly available computer (MacBook Air Retina 13-inch 2019, with a 1.6 GHz Dual-Core Intel Core i5 processor and 8 GB of RAM, Apple Inc. Cupertino, CA, USA). As with the training cohort, CXRs from both validation cohorts were resized and normalized at the time of processing by the CNN. Each CXR was rapidly processed by the CNN with a processing time to make a prediction of pneumonia of 129 ± 36 ms (mean ± standard deviation) for PneumOld-CT and of 141 ± 33 ms for PACSCAN. For each CXR, the output of CNN was a binarized prediction of pneumonia (1 = pneumonia; 0 = no pneumonia), with a percent probability.

### Performance comparison between readers and imaging modalities

Statistical comparisons between the different CXR readers (CNN, emergency physicians, or radiologists) and the two imaging modalities (CXR or CT) were performed separately for the two validation cohorts to assess the consistency of the results. For all predictions, we used the reference diagnosis as previously described.

The ROC-AUC was used to assess accuracy of clinicians, senior radiologists, and AI on CXR and for radiologists on CT. These ROC-AUC were computed based on Likert-score reported by clinicians for CXR) and radiologists for CXR and C), as described above, and on the probabilistic output produced by the CNN for AI. ROC-AUCs were compared using two-tailed DeLong test. Accuracy, sensitivity, specificity, positive predictive value (PPV), negative predictive value (NPV), positive likelihood ratio, negative likelihood ratio, and diagnostic odds ratio (DOR) were reported separately for the two cohorts with their 95% confidence intervals. Confidence intervals were computed using Wilson’s method, which does not rely on a normal approximation and results in accurate confidence intervals even for small sample sizes [29, 30].

## Results

**Fig. 1:**
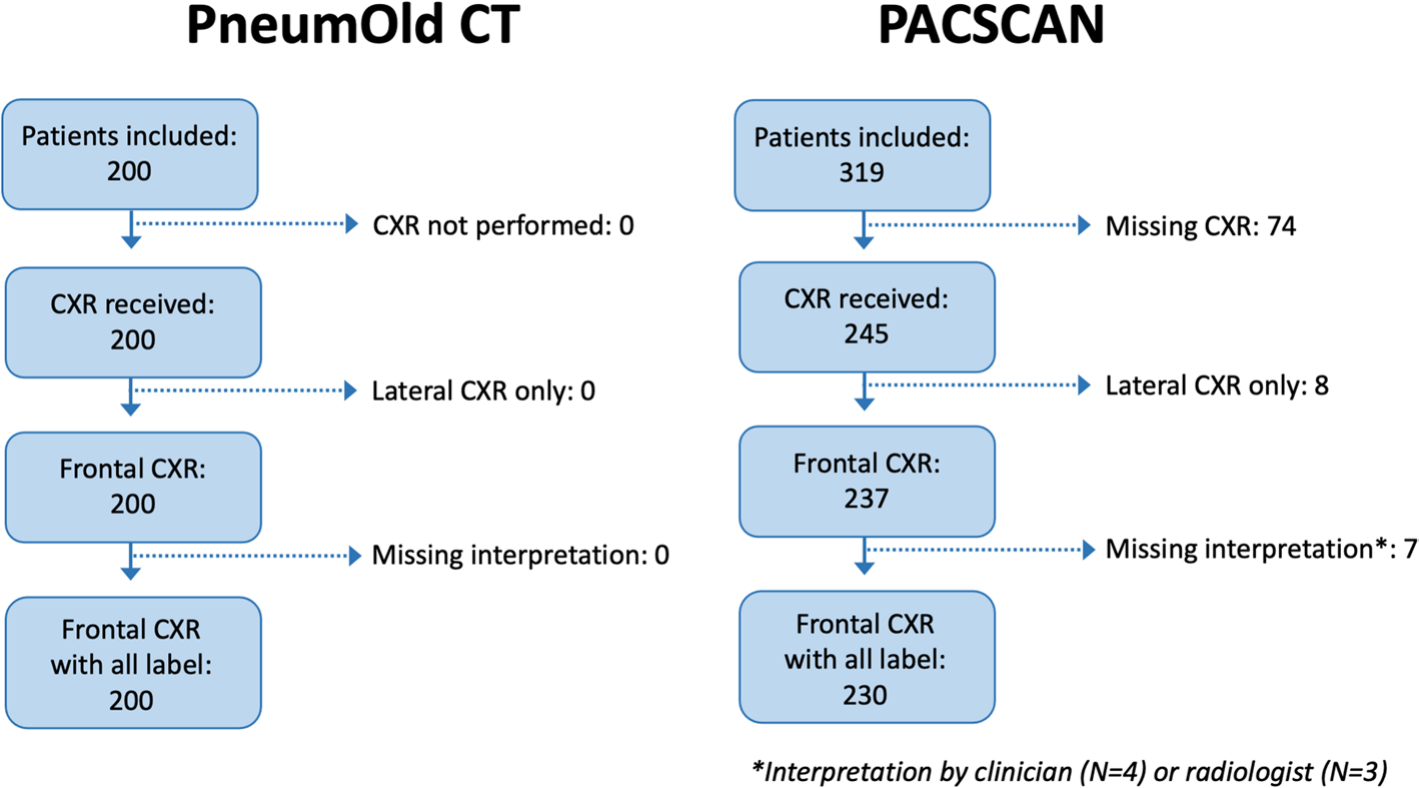
Flowchart of the PneumOld-CT and PACSCAN cohorts

All the 200 CXRs from the PneumOld-CT study and 230 of 319 CXRs from the PACSCAN study were available (Fig. 1). The lower inclusion rate of CXRs for the PACSCAN study was due to missing data related to their storage on digital media and then manual retrieval, whereas CXRs from the PneumOld-CT study were stored on a research picture archiving and communication system, so directly accessible to the authors. Demographic characteristics of patients in both cohorts are reported in the original articles [26, 27] and in Table 1 of the current manuscript.

**Table 1:** Main characteristics and outcomes of patients included in the validation cohorts

	**PneumOld-CT (*N* = 200)**	**PACSCAN (*N* = 230)**
Mean age (years)	84 (79–90)^a^	65 (20)
Sex (% woman)	98 (49)	164 (51)
Chronic obstructive pulmonary disease	35 (18)	64 (20)
Chronic heart failure (%)	103 (52)	39 (12)
Cough	170 (85)	240 (76)
Sputum production	74 (37)	147 (46)
Hypoxemia (SaO_2_ < 90% on room air)	102 (51%)	49 (17)
Fever	116 (58)	112 (35)
Pneumonia confirmed (%)	133 (67)	163 (51)
Severe pneumonia (PSI cat IV-V, or CURB-65 ≥ 3)	89 (45)	118 (37)
30-day death (%)	11 (5)	13 (4)

*CURB-65*, confusion, urea > 7 mmol/L, respiratory rate > 30/min, blood pressure < 90 mmHg, age > 65 years. *PSI*, Pneumonia severity index. Data are mean with standard deviation or frequencies with percentage except when marked otherwise

^a^Median with interquartile range

Diagnostic testing accuracy measures for clinicians, senior radiologists, and AI on CXR and for radiologists on CT are reported in Table 2. Consistently in both cohorts, accuracy for the diagnosis of pneumonia was higher for CT scan (89.6% in PACSCAN and 79.0% PneumOld-CT) than for CXR, whoever was the assessor. Radiologist 1 showed an accuracy of 70.9% (PACSCAN) and 75.1% (PneumOld-CT), radiologist 2 of 71.0% (in PneumOld-CT), the AI model 72.6% and 71.0%, and emergency physicians 63.0% and 64.0%, respectively. So, the radiologists and AI had close diagnostic accuracy on CXRs (between 70.9 and 75.5%), and their accuracy was higher than that of emergency physicians (see Table 2).

**Table 2:** Diagnostic testing accuracies

	Accuracy	Sensitivity	Specificity	PPV	NPV	LR +	LR-	DOR
PneumOld-CT cohort								
Clinicians (CXR)	64.0% (57.1–70.3)	76.7% (68.8–83.1)	38.8% (28.0–50.8)	71.3% (63.4–78.1)	45.6% (33.4–58.4)	1.253 (0.956–1.688)	0.601 (0.334–1.112)	2.087
Radiol. 1 (CXR)	75.5% (69.1–80.9)	69.9% (61.7–77.1)	86.6% (76.4–92.8)	91.2% (84.1–95.3)	59.2% (49.3–68.4)	5.206 (2.613–10.658)	0.347 (0.247–0.502)	14.983
Radiol. 2 (CXR)	71.0% (64.4–76.8)	67.7% (59.3–75.0)	77.6% (66.3–85.9)	85.7% (77.8–91.1)	54.7% (44.7–64.4)	3.023 (1.760–5.335)	0.417 (0.291–0.614)	7.256
AI (CXR)	71.0% (64.4–76.8)	74.4% (66.4–81.1)	64.2% (52.2–74.6)	80.5% (72.6–86.5)	55.8% (44.7–66.4)	2.078 (1.390–3.193)	0.398 (0.253–0.643)	5.217
LDCT	79.0% (72.8–84.1)	72.9% (64.8–79.8)	91.0% (81.8–95.8)	94.2% (87.9–97.3)	62.9% (53.0–71.8)	8.144 (3.563–19.131)	0.297 (0.211–0.430)	27.394
PACSCAN cohort								
Clinicians (CXR)	63.0% (56.6–69.0)	61.4% (53.2–69.1)	65.6% (55.3–74.6)	73.5% (64.9–80.7)	52.2% (43.1–61.2)	1.783 (1.189–2.715)	0.588 (0.415–0.847)	3.031
Radiol. (CXR)	70.9% (64.7–76.4)	67.9% (59.7–75.0)	75.6% (65.8–83.3)	81.2% (73.2–87.2)	60.2% (51.0–68.7)	2.776 (1.744–4.484)	0.425 (0.300–0.612)	6.525
AI (CXR)	72.6% (66.5–78.0)	72.1% (64.2–78.9)	73.3% (63.4–81.4)	80.8% (73.0–86.7)	62.9% (53.3–71.5)	2.705 (1.753–4.237)	0.380 (0.259–0.565)	7.122
CT (LDCT/full–dose)	89.6% (84.9–92.9)	87.1% (80.6–91.7)	93.3% (86.2–96.9)	95.3% (90.2–97.8)	82.4% (73.8–88.5)	13.071 (5.844–29.67)	0.138 (0.086–0.225)	94.889

Results are reported in percent along with their 95% confidence intervals. The radiologists in both cohorts are senior radiologists specialized in thoracic imaging. *AI*, Artificial intelligence; *CT*, Computed tomography; CXR, Chest-x-ray; *LDCT*, Low-dose computed tomography; *LR* + , Positive likelihood ratio; *LR-*, Negative likelihood ratio; *DOR*, Diagnostic odd radio; *PPV*, Positive predicting value; *NPV*, Negative predicting value; *Radiol.*, Radiologist; *ROC-AUC*, Area under the receiver operating characteristic curve

In both cohorts, all CXR and CT reviewers had higher PPVs than NPVs for identifying patients with pneumonia. PPVs were relatively close between both cohorts, but NPVs were higher for all reviewers in the PACSCAN study.

DORs for the diagnosis of pneumonia were excellent for CT in both cohorts (94.9 for PACSCAN and 27.4 for PneumOld-CT). They were excellent for one radiologist on CXR (15.0 in PneumOld-CT) and good for the other (6.5 in PACSCAN and 7.3 in PneumOld-CT). The DOR of the AI model on CXR was relatively close to the radiologists (7.1 and 5.2 in PACSCAN and PneumOld-CT, respectively). The emergency physicians DOR on CXR was lower (3.0 and 2.1, respectively). Details of positive and negative likelihood ratios are reported in Table 2.

Statistical comparison of the performance of clinicians and radiologists on CXR, and radiologists on CT are reported in Table 3 and 4. Consistently in both cohorts, radiologists and AI had significantly higher ROC-AUCs than clinicians for pneumonia diagnosis on CXRs. We did not observe any significant difference in ROC-AUC between radiologists and AI. However, all CXR readers (radiologists, AI, and emergency physicians) had significant lower performance compared with CT readers, with the exception of one senior radiologist in the PneumOld-CT cohort.

**Table 3:** ROC-AUC and statistical comparisons of diagnostic performances (PneumOld-CT cohort)

		Clinicians	Radiologist 1	Radiologist 2	AI	LDCT
	ROC-AUC	0.577 (0.509–0.646)	0.782 (0.726–0.839)	0.726 (0.662–0.791)	0.738 (0.664–0.812)	0.820 (0.769–0.871)
Clinicians	0.577 (0.509–0.646)					
Radiol. 1	0.782 (0.726–0.839)	*p* < 0.001				
Radiol. 2	0.726 (0.662–0.791)	*p* < 0.001	*p* = 0.134			
AI	0.738 (0.664–0.812)	*p* < 0.001	*p* = 0.269	*p* = 0.768		
LDCT	0.820 (0.769–0.871)	*p* < 0.001	*p* = 0.233	*p* = 0.013	*p* = 0.065	

ROC-AUC results are reported in percent along with their 95% confidence interval. Statistical comparison of two sets of predictions by area under the receiver operating characteristic curve using method from Sun and Xu. [reference #[31]. *AI*, Artificial intelligence; *CT*, Computed tomography; *LDCT*, Low-dose computed tomography; *ROC-AUC*, Area under the receiver operating characteristic curve

**Table 4:** ROC-AUC and statistical comparisons of diagnostic performances (PACSCAN cohort)

		Clinician	Radiologist	AI	CT
	ROC-AUC	0.635 (0.571–0.699)	0.717 (0.658–0.776)	0.735 (0.667–0.802)	0.919 (0.884–0.954)
Clinicians	0.635 (0.571–0.699)				
Radiologist	0.717 (0.658–0.776)	*p* = 0.022			
AI	0.735 (0.667–0.802)	*p* = 0.021	*p* = 0.683		
CT	0.919 (0.884–0.954)	*p* < 0.001	*p* < 0.001	*p* < 0.001	

ROC-AUC results are reported in percent along with their 95% confidence interval. Statistical comparison of two sets of predictions by area under the receiver operating characteristic curve using method from Sun and Xu. [reference #[31]. *AI*, Artificial intelligence; *CT*, Computed tomography; *LDCT*, Low-dose computed tomography; *ROC-AUC*, Area under the receiver operating characteristic curve

**Fig. 2:**
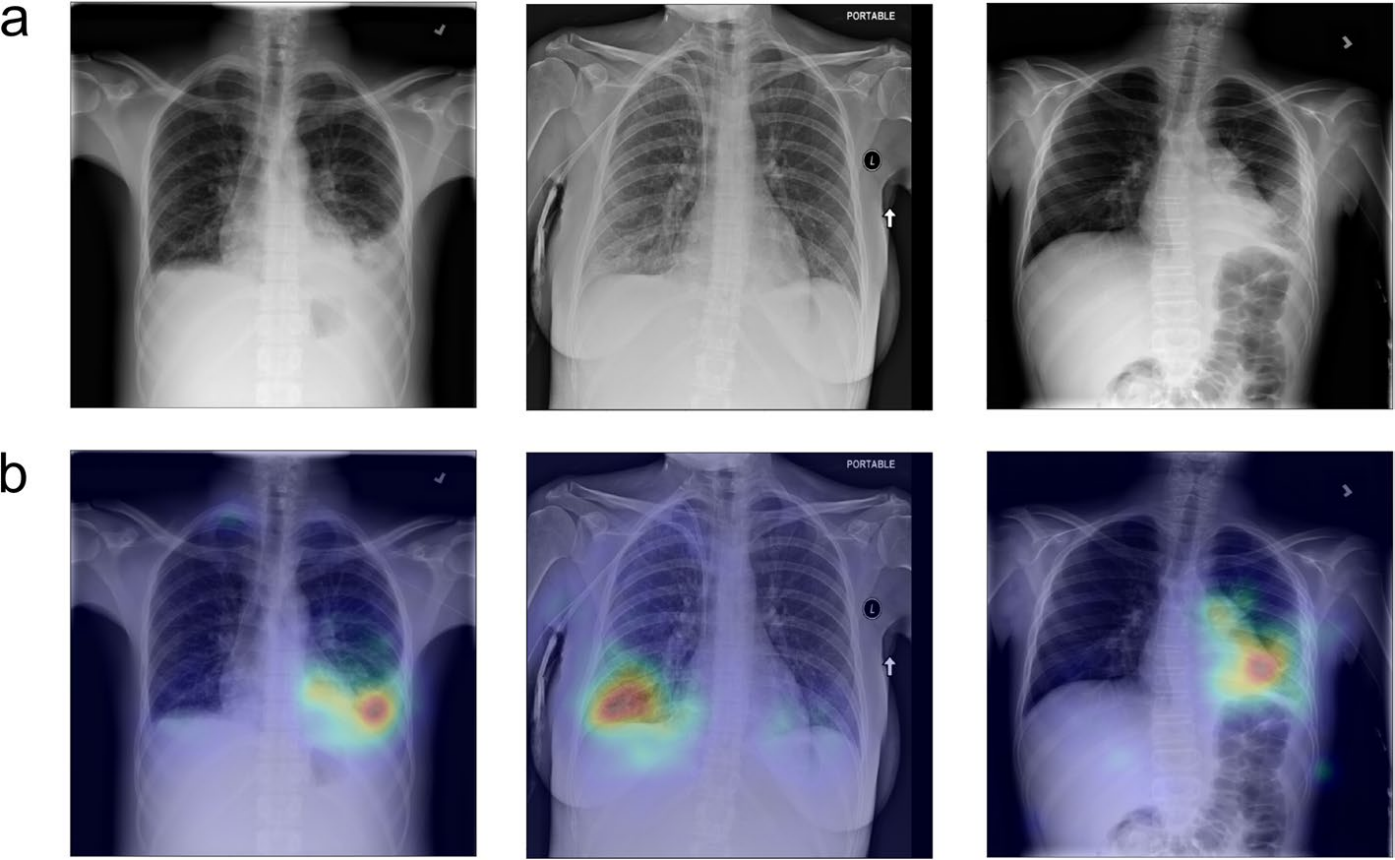
Three illustrative cases of chest x-ray of patients with pneumonia (**a**, upper row) and their relative saliency maps using gradient-weighted class activation mapping (**b**, lower row)

In Fig. 2, three cases of patients with pneumonia visible on CXRs and their saliency maps generated by the CNN are shown.

When repeating data analysis for the PACSCAN cohort, considering possible pneumonia as part of the group of patients with a final diagnosis of pneumonia, we observed close results. However, the performance of clinicians and radiologists on CXRs is more variable than that of AI (Additional file 1: Supplementary Table 1). The performance of radiologists even becomes statistically inferior to that of AI and is no longer statistically inferior to that of clinicians. Overall, however, CXR reviewers (clinicians, radiologists, and AI) continue to perform worse than CT reviewers (Additional file 1: Supplementary Table 2).

## Discussion

Our study reports that the performance of a CNN model compared to a robust reference diagnosis is inferior to that previously described when compared only to radiologists’ report. This suggests that the performance of AI reported so far has been overestimated because of a comparison with an insufficiently reliable reference diagnosis. Our study also found that the performance of a CNN model in diagnosing pneumonia on CXR was similar to that of senior thoracic radiologists and significantly better than that of emergency physicians. Furthermore, AI diagnosis on CXR was inferior to a diagnosis made by radiologists on CT.

Our study highlights the importance of an appropriate reference diagnosis in studies evaluating the clinical utility of AI tools. Previous studies evaluating the performance of AI for the diagnosis of pneumonia have used the radiologists’ diagnosis as a reference test. They have shown excellent performances, up to a ROC-AUC of 0.99 reported in a recent meta-analysis [32]. However, the reference diagnosis in these studies is far from optimal. First, the definition of pneumonia requires both clinical findings and the presence of an acute infiltrate on an imaging modality. Radiologist’s diagnosis does not always incorporate clinical data. Secondly, due to intrinsic limitations of CXR, and as demonstrated by other authors, radiologist diagnosis has limited reproducibility [6, 10]. Moreover, in most of datasets, annotation methods are heterogenous and not specifically addressing the pneumonia diagnosis. Third, incorporation bias (*i.e.*, the index test, here CNN interpretation of a CXR, is also a central part of the reference standard) is an unresolved issue.

We tried to surpass these limitations by using an expert consensus based on a vast array of clinical, biological, and radiological information, which represents the best achievable reference diagnosis in a clinical setting, hence providing a more unbiased estimate of the performance of AI. The corresponding ROC-AUC was relatively low, *i.e.*, 0.74.

An additional feature of our study may explain the lower-than-expected performance of CNN: CXRs were obtained in real-world conditions, with associated technical challenges to obtain high-quality studies. This translates in approximately 60% of CXRs in both cohorts obtained on bedridden patients, which is representative of the management of these patients. This may affect the ability of all readers to identify pneumonia but is more representative of the real diagnostic potential of CXR in this setting.

CNN and senior radiologists had close diagnostic performance when interpreting CXR, which was significantly better than that of emergency physicians. We found some heterogeneity between radiologists in their accuracy, as one of the two radiologists from PneumOld-CT showed significantly better metrics than the other one. The accuracy of our CNN was similar in the two cohorts, suggesting good generalizability. In an emergency setting with limited timely access to senior radiologists, AI could therefore assist clinicians in the interpretation of CXR. However, future prospective studies are needed to validate this hypothesis.

The diagnostic performance of CXR consistently remained inferior to CT scan, regardless of the reader (clinicians, radiologists, or CNN). Our study thus confirms that CT has a significantly better DOR than CXR and thus may play an important role in the diagnostic workup of pneumonia depending on the clinical situation, as previously proposed by other authors [5, 33, 34]. It is noteworthy that CT performance was better for the PACSCAN cohort. This difference may arise from (i) the fact that CT scans in the PACSCAN study could be full-dose CT and/or contrast-enhanced if necessary and (ii) that the patients included in the PACSCAN cohort were younger than those of the PneumOld-CT cohort. Indeed, the interpretation of thoracic imaging has been described to be more challenging in the elderly population [35–37].

The reference diagnosis (*i.e.*, the “label” in AI studies) of the training data in our model was that given by radiologists, whose limitations were raised earlier. Thus, one arising question is whether the AI could achieve better performance by being trained with a better label (*i.e.*, stronger reference diagnosis). However, the difficulty of obtaining such a training dataset with a strong reference diagnosis of sufficient size did not allow us to test this hypothesis.

Our CNN model was developed based on an open access CNN architecture and trained with publicly available data. The accessibility of our model’s development should therefore encourage the scientific community to continue to share the methods and data needed to validate AI tools on clinical cohorts. One of the strengths of this work is that, unlike many studies evaluating the performance of AI in the diagnosis of pneumonia on CXR, the reference diagnosis was not that of radiologists but adjudicated a posteriori by a panel of experts using all available information.

We identify several limitations to our study. First, due to technical issues independent of patients’ characteristics, we were not able to reanalyze all the PACSCAN patients. Second, the diagnosis by CNN in our study was based only on frontal CXRs and did not integrate clinical, demographic, and biological data pertinent for the diagnosis of pneumonia. If used in a clinical setting, a similar AI tool could be an aid to the interpretation of radiological studies but should not substitute to the physician diagnosis. Third, we did not assess the impact of heart failure, pleural effusion, cavitation, and lung mass on the accuracy of interpretation of the DL model. Fourth, our algorithm did not distinguish between bacterial and viral pneumonia, which has an impact on patient management.

In conclusion, our study highlights the importance of a strong reference diagnosis to avoid overestimating the performance of AI models. When compared to a multimodal reference diagnosis, the accuracy of AI in diagnosing pneumonia on CXR was similar to that of expert radiologists but lower than that previously reported in the literature. This difference may be due to the validation of AI against the diagnosis of radiologists in previous studies, despite its limited sensitivity. Finally, although we found the diagnostic performance of CXR to be inferior to CT, regardless of reader, AI was more accurate than emergency physicians and may therefore have a role in assisting with CXR interpretation when an expert radiologist is not readily available.

### Supplementary Information

**Additional file 1:** **Appendix 1.** Reference diagnosis.

## Data Availability

The datasets used during the current study are available from the corresponding author on reasonable request. The deep learning model developed in this study is available here: https://github.com/jeremyhofmeister/pneumoniaCXR.

## Abbreviations

AI: Artificial intelligence
CNN: Convolutional neural network
CT: Computed tomography
CXR: Chest x-ray
DL: Deep learning
DOR: Diagnostic odds radio
NPV: Negative predictive value
PACSCAN: “Early Chest Computed Tomography Scan to Assist Diagnosis and Guide Treatment Decision for Suspected Community-Acquired Pneumonia” study (NCT 01574066)
PneumOld-CT: “Low-dose CT for the diagnosis of pneumonia in elderly patients” study (NCT 02467192)
PPV: Positive predictive value
ROC-AUC: Area under the receiver operating characteristic curve
