# Supplementary material for: Validating the accuracy of deep learning for the diagnosis of pneumonia on chest x-ray against a robust multimodal reference diagnosis: a post hoc analysis of two prospective studies
Source: Eur Radiol Exp. 2024 Feb 2;8:20. doi: 10.1186/s41747-023-00416-y (PMC10834924; doi:10.1186/s41747-023-00416-y)
Supplement: Supplementary file 1 — Additional file 1: Appendix 1. Reference diagnosis. [file 41747_2023_416_MOESM1_ESM.docx]

**Validating the accuracy of deep learning for the diagnosis of pneumonia on chest x-ray against a robust multimodal reference diagnosis: a post-hoc analysis of two prospective studies**

**ELECTRONIC SUPPLEMENTARY MATERIAL**

**Appendix 1: Reference Diagnosis**

**PneumOldCT cohort**

In the Geneva cohort (PneumOldCT), patients were enrolled mainly with suspected community-acquired pneumonia (CAP) (N=162, 81%), some with nursing home-acquired pneumonia (NHAP) (N=22, 11%) and hospital-acquired pneumonia (onset of symptoms >72h after hospitalization) (N=16, 8%).

At the end of the study, an adjudication committee blinded to the LDCT scan results retrospectively assessed the likelihood of pneumonia based on all other available patient data: CXRs, biological and microbiological data, including the final medical report, but with all references to the LDCT results removed.

Clinical data were cough, chest pain, sputum production, dyspnea, crackles, chills, fever, confusion, fall, increased respiratory and heart rate, decreased systolic and diastolic blood pressure. Biologic data were white blood cell count, procalcitonin, C-reactive protein, urea, pH, PaO2 or SaO2, and microbiologic data if positive (culture of blood, urine, sputum, pleural effusion, and urine antigen, viral PCR on nasopharyngeal swabs).

Eleven board-certified specialists in infectious diseases, pulmonology, internal medicine, and radiology served on the committee. All were senior attending physicians with expertise in the care of patients with pneumonia. Each patient's diagnosis of pneumonia was analyzed by the adjudication committee as follows: first, each expert gave an individual opinion of the likelihood of the patient having pneumonia on a five-point Likert scale (excluded, low, medium, high, certain); second, each expert re-examined the cases on which the committee had disagreed, with full knowledge of the other experts' initial decisions; finally, in plenary session and in the presence of a radiologist, the adjudication committee reached consensus decisions on cases that remained unresolved after the first two phases. The final decision of the adjudication committee was considered the reference diagnosis. The agreement rate between the adjudication committee experts was assessed. Their probability of diagnosing pneumonia was compared with the pre-LDCT probability.

For our actual work, the adjudication committee reassessed their diagnostic probability using the LDCT results (as was done in the PACSCAN cohort).

**PACSCAN cohort**

In the French cohort (PASCAN), only patients with suspected CAP were included.

Clinical data were cough, chest pain, sputum production, dyspnea, crackles, chills, headache, myalgia, fever, confusion, increased respiratory and heart rate, decreased systolic and diastolic blood pressure. Biological data were white blood cell count, procalcitonin, C-reactive protein, urea, pH, PaO_2_, or SaO_2_.

The adjudication committee consisted of three independent experts in infectious diseases, pneumology, and radiology within a panel of nine experts who were masked to the assessment of the emergency investigators. For each patient, the adjudication committee assigned two diagnostic probabilities. First, the adjudication committee retrospectively assigned the probability of a CAP diagnosis using a four-point Likert scale based on data collected in the baseline standardized case report forms, images of radiographs, and a multidetector CT scan recorded on a special DVD (hereafter referred to as "adjudication committee CAP probability after CT scan"). Second, the adjudication committee assigned a final probability of CAP diagnosis using all available follow-up data, including the patient's discharge summary and a telephone follow-up by the adjudication committee investigators with the patient, family, or primary care physician at day 28 (hereafter referred to as "day 28 adjudication committee CAP probability"). For patients lost to follow-up, the CAP adjudication committee classification after the CT scan was used. This Day 28 adjudication was used as the gold standard in the study.

**Supplementary Table 1. Diagnostic testing accuracies with lower threshold for pneumonia, in the PACSCAN cohort**

|  | **Accuracy** | **Sensitivity** | **Specificity** | **PPV** | **NPV** | **LR+** | **LR-** | **DOR** |
| --- | --- | --- | --- | --- | --- | --- | --- | --- |
| **Clinician (on CXR)** | 56.1% (49.6-62.3) | 95.8% (90.5-98.2) | 14.3% (9.0-22.0) | 54.1% (47.3-60.7) | 76.2% (54.9-89.4) | 1.117 (0.994-1.258) | 0.297 (0.083-1.061) | 3.767 |
| **Radiol. (on CXR)** | 57.4% (50.9-63.6) | 94.9% (89.3-97.6) | 17.9% (11.9-26.0) | 54.9% (48.0-61.6) | 76.9% (57.9-89.0) | 1.155 (1.014-1.319) | 0.285 (0.090-0.897) | 4.058 |
| **AI (on CXR)** | 69.1% (62.9-74.7) | 72.9% (64.2-80.1) | 65.2% (56.0-73.4) | 68.8% (60.2-76.3) | 69.5% (60.2-77.5) | 2.093 (1.459-3.007) | 0.416 (0.271-0.639) | 5.030 |
| **CT (LDCT or full dose)** | 83.0% (77.7-87.3) | 99.2% (95.4-99.9) | 66.1% (56.9-74.2) | 75.5% (68.1-81.6) | 98.7% (92.8-99.8) | 2.922 (2.212-3.867) | 0.013 (0.002-0.082) | 227.842 |

Results are reported in percent along with their 95% confidence interval. The radiologists in both cohorts are senior radiologists specialized in thoracic imaging. Abbreviations: AI: artificial intelligence, CT: computed tomography, LR+: positive likelihood ratio, LR-: negative likelihood ratio, DOR: diagnostic odd radio, PPV: positive predicting value, NPV: negative predicting value ROC-AUC: area under the receiver operating characteristic curve.

**Supplementary Table 2. ROC-AUC and statistical comparisons of diagnostic performances with lower threshold for pneumonia, in the PACSCAN cohort**

|  |  | **Clinician** | **Radiologist** | **AI** | **CT** |
| --- | --- | --- | --- | --- | --- |
|  | **ROC-AUC** | 0.635 (0.571-0.699) | 0.564 (0.523-0.605) | 0.663 (0.591-0.736) | 0.830 (0.786-0.874) |
| **Clinician** | 0.550 (0.513-0.588) |  |  |  |  |
| **Radiologist** | 0.564 (0.523-0.605) | p = 0.601 |  |  |  |
| **AI** | 0.663 (0.591-0.736) | p = 0.005 | p = 0.008 |  |  |
| **CT** | 0.830 (0.786-0.874) | p < 0.001 | p < 0.001 | p < 0.001 |  |

ROC-AUC results are reported in percent along with their 95% confidence interval. Statistical comparison of two sets of predictions by area under the receiver operating characteristic curve using method from Sun and Xu.(35) Abbreviations: AI: artificial intelligence, CT: computed tomography, LDCT: low-dose computed tomography, ROC-AUC: area under the receiver operating characteristic curve.
